# Research on Surface Tracking and Constant Force Control of a Grinding Robot

**DOI:** 10.3390/s23104702

**Published:** 2023-05-12

**Authors:** Xiaohua Shi, Mingyang Li, Yuehu Dong, Shangyu Feng

**Affiliations:** 1 School of Mechanical Engineering, Yanshan University, Qinhuangdao 066000, China; qqxr1998@126.com (Y.D.); fengshangyu@inovance.com (S.F.); 2 School of Vehicle and Energy, Yanshan University, Qinhuangdao 066000, China; limingyang1998@126.com; 3 Suzhou Huichuan Control Technology Co., Ltd., Suzhou 215000, China

**Keywords:** grinding robot, constant force control, surface tracking, fuzzy PID control, wind turbine blade

## Abstract

To improve the quality and efficiency of robot grinding, a design and a control algorithm for a robot used for grinding the surfaces of large, curved workpieces with unknown parameters, such as wind turbine blades, are proposed herein. Firstly, the structure and motion mode of the grinding robot are determined. Secondly, in order to solve the problem of complexity and poor adaptability of the algorithm in the grinding process, a force/position hybrid control strategy based on fuzzy PID is proposed which greatly improves the response speed and reduces the error of the static control strategy. Compared with normal PID, fuzzy PID has the advantages of variable parameters and strong adaptability; the hydraulic cylinder used to adjust the angle of the manipulator can control the speed offset within 0.27 rad/s, and the grinding process can be carried out directly without obtaining the specific model of the surface to be machined. Finally, the experiments are carried out, the grinding force and feed speed are maintained within the allowable error range of the expected value, and the results verify the feasibility and effectiveness of the position tracking and constant force control strategy in this paper. The surface roughness of the blade is maintained within Ra = 2~3 μm after grinding, which proves that the grinding quality meets the requirements of the best surface roughness required for the subsequent process.

## 1. Introduction

The development of the wind power industry is of great significance to environmental protection, and there has been rapid development, with increasing requirements for wind turbines in recent years [1]. Therefore, the manufacturing process of wind turbine blades and other components is gradually becoming more intelligent and automated [2], and the process of grinding wind turbine blades is essential. The roughness after grinding affects the blade’s subsequent effectiveness, and the grinding process itself determines the production cost and service life of the blade. Due to the irregular shape and large size of a wind turbine blade’s surface, artificial grinding, which is currently the most common method of treatment, is not merely inefficient, but also struggles to achieve uniformity, and polished glass fiber dust can cause environmental pollution and damage to workers’ health [3,4]. In consequence, the design of, and research into, wind turbine blade grinding robots has gradually attracted more interest in recent years. Some of them employ a gantry structure [5,6] equipped with a grinding head on each side, whereby the gantry moves along a guide rail to perform the grinding of the blades. This model achieves high grinding efficiency but requires much space. Others use mobile platforms equipped with multi-degree-of-freedom manipulators and grinding devices [7], which can effectively adapt to the site, but with more difficult manipulator control.

With the gradual increase in industrial automation in recent years, the requirements relating to the control and repetitive positioning accuracy of robots have increased, and the environmental aspects of robots need to be improved. The surfaces of wind turbine blades are complex, with curvature variation in the axial and circumferential directions; thus, the control of the grinding robot is particularly important. To ensure a suitable grinding effect, it is essential to maintain a constant grinding force and the real-time tracking of the blade surface.

Most previous studies have focused on force control, which can be divided into passive control and active control [8]. Guo et al. [9] proposed a new two-dimensional adjustable force mechanism that does not require additional sensors. However, because of the low accuracy and poor stability of this method of passive constant force control, active force control has become a research hotspot.

Active force control includes impedance control and force–position hybrid control. Shen et al. [10] proposed a fuzzy-based impedance control algorithm, with which the contact force error could be controlled within 2N for the external environment with unknown stiffness. Wahballa et al. [11] used an OSRDF impedance controller to offset the damping force and the contact force tracking error, which improved the system stability.

PID control is a common method applied to achieve active force control. The process is proportional, integral and differential, enabling quick responsiveness and high stability in control, and the robustness of the mode of PID control is strong. To resolve the issues of model construction and algorithm complexity that arise in the active flexibility control method, Yao et al. [12] proposed a fuzzy PID smooth grinding control algorithm based on a two-degree-of-freedom constant force grinding device driven by pneumatic force, wherein the position and force are decoupled and controlled separately, and verified that the control precision and response time of the fuzzy PID control algorithm were better than those of the conventional PID control method. In addition, Sun et al. [13] confirmed the effectiveness of the fuzzy PID control algorithm. Dai et al. [14] also presented a backstepping + PID control method to closely track the applied force.

As such, many scholars use PID as the control strategy in their research. Xu et al. [15] proposed the combination of an active strategy, based on PI/PD control, and a passive strategy, based on PID control, which was shown to improve the accuracy and efficiency of the controlled force and avoid over- and under-cutting. Zhang et al. [16] presented a method for optimizing the parameters of a robot PD constant grinding force controller using deep reinforcement learning DRL Rainbow, which can ensure that a constant force is applied during grinding by adjusting the grinding depth. Further, some studies have been carried out on the normal vector of the surface; in establishing the positioning of the grinding tool during processing, Zhao et al. [17] proposed an adaptive PD constant force controller and a normal vector search algorithm, which together ensure that constant force is applied during the grinding of workpieces with unknown shapes. Wang et al. [18] put forward a force–position hybrid control method based on a PD constant force control algorithm. Han et al. [19] presented a fuzzy gain scheduling PID controller, which could reduce the influence of dynamic characteristics on the control system.

As regards the influence of curvature variance, beginning with an improved material removal model, Li et al. [20] took the material to be removed from different positions on the object being machined as the research object, and constructed a hybrid force–position control algorithm to ensure the contour accuracy of workpieces with complex curved surfaces.

Some scholars have proposed other new algorithms to ensure constant force control. In order to resolve the shortcomings of traditional robots’ constant force output, Jia et al. [21] combined the PID control method with a particle swarm optimization algorithm (PSO) to realize the constant force output of a cylinder and drive the grinding device. Zhao et al. [22] developed a strategy for constant force grinding that reconstructs the surface of the workpiece based on the point cloud data obtained via visual measurements, without requiring an accurate CAD model of the workpiece. In the grinding process, noise in the machining environment will affect the constant force control of the robot. Dai et al. [23] proposed a new force control method using an extended state observer (ESO) that can reduce fluctuations in the grinding force in the normal direction. Due to the impact between the grinding tool and the surface being machined, there is constant fluctuation in the contact force, and so Xiao et al. [24] put forward an active disturbance rejection method. Here, in order to achieve constant force control during machining, an RBF neural network model and an iterative algorithm are combined. Zhang [25] presented a variable-gain iterative learning force–position hybrid control method with ideal contour force as the contact point processing idea, which could still ensure the control accuracy of contour force when the tracking error was not accurate enough. Li et al. [26] used the force–position hybrid control method to estimate and adjust the contact state between the robot and the unknown workpiece, which could provide a constant normal contact force. Zhang et al. [27] proposed a constant force grinding controller based on proximal strategy optimization, which improved the response ability of the system.

The roughness of the outer surface of the wind turbine blade before grinding is about Ra = 0.8~1.5 μm, and the requirements are met when this value after grinding is Ra = 1.5~8 μm. A roughness of Ra = 2.0~3.0 μm yields the best results in the subsequent painting of the blade.

In this paper, a wind-turbine-blade-grinding robot, along with its control strategy and related algorithms, are introduced. Given the size and complexity of the surfaces of wind turbine blades, this paper considers the surface tracking and constant force control of blades simultaneously, so as to enhance the surface quality of wind turbine blades after grinding. Firstly, the structure of the grinding robot is described, and the method for planning the motion of the robot is designed. Then, the strategies of surface tracking and constant force control are established; fuzzy PID control is applied to the power source of the manipulator, and gravity compensation for the grinding robot is conducted. Finally, the controlling algorithm constructed in this paper is applied to the operation of a grinding robot to validate the reliability of our method of surface tracking and constant force control; by comparison with artificial grinding, the feasibility of the effectiveness of the scheme is confirmed. The experimental results are discussed at the end of the paper.

## 2. Grinding Robot

### 2.1. Structure of the Grinding Robot

**Figure 1:**
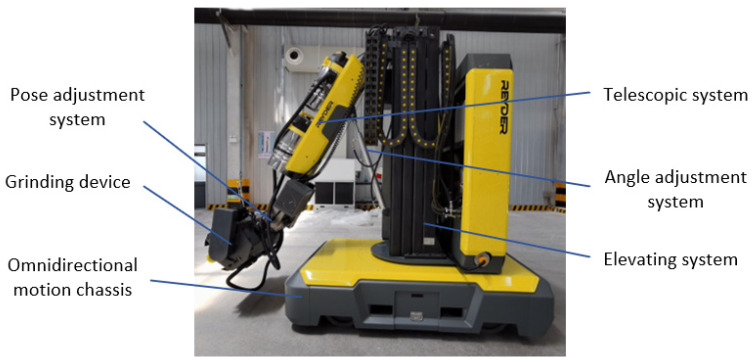
Grinding robot.

The grinding robot addressed in this paper is composed of three main parts: an omnidirectional motion chassis, a five-degree-of-freedom manipulator and a flexible grinding device, as shown in Figure 1. The omnidirectional motion chassis is equipped with a five-degree-of-freedom manipulator to enable the maneuverability of the grinding robot, and a flexible grinding device is installed at the end of the manipulator.

**Figure 2:**
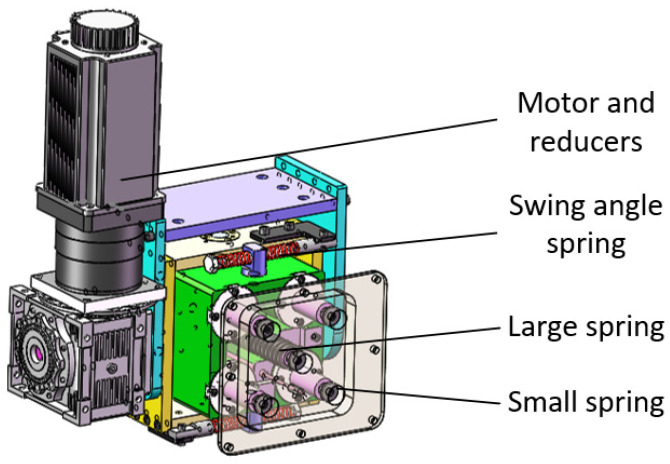
Pose adjustment system.

The structure of the pose adjustment system of the flexible grinding device is shown in Figure 2. Four small springs are arranged around the directional connecting block in the pose adjustment system to ensure that the flexible grinding device can move around the axial surface of the blade, and a micro displacement sensor is included under the large spring in the center, so the normal force of the grinding device on the blade is governed by measuring the compression distance of this spring.

### 2.2. Motion Planning and Control Scheme

**Figure 3:**
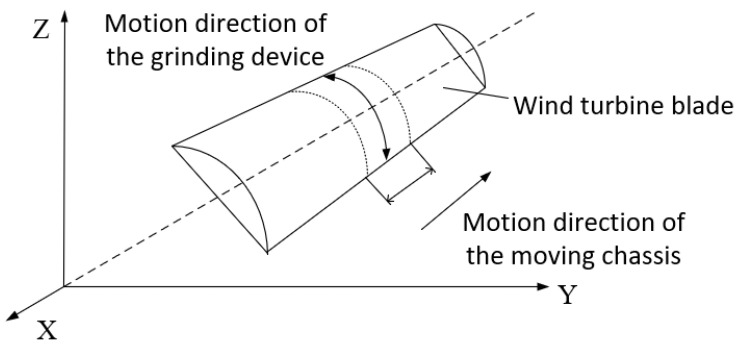
Motion planning of the grinding robot.

Unlike the structure of a traditional manipulator, the wind-turbine-blade-grinding robot addressed in this paper is a type of vehicle-mounted combined manipulator. For simplicity, the control of the total motion of the grinding robot is separated into two steps. First is the position adjustment of the whole machine; the grinding robot is moved to the correct area for blade processing in this stage. Here, only the control of the moving chassis is considered, while the control of the manipulator is not considered. Second is the grinding stage, during which the mechanical arm maneuvers the front-end grinding device to carry out the grinding of the blade. At this stage, the moving chassis is stationary, and only the control of the motion and force of the mechanical arm are considered, so as to ensure the surface tracking of, and the control of force applied to, the blade. After completing a given grinding area, the above stages are repeated in the next area. In this paper, a longitudinal-spacing grinding method is adopted, and the planning of the overall motion of the grinding robot is illustrated in Figure 3.

**Figure 4:**
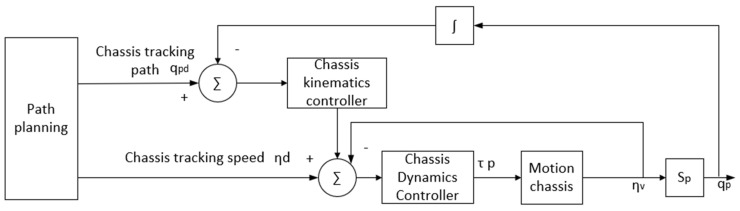
Strategy of controlling the grinding robot in the position-adjustment stage.

In the first stage, only the position of the moving chassis needs to be adjusted, and must be kept at a distance of 1.5~2.5 m from the blade. Figure 4 shows the control method employed in this stage.

**Figure 5:**
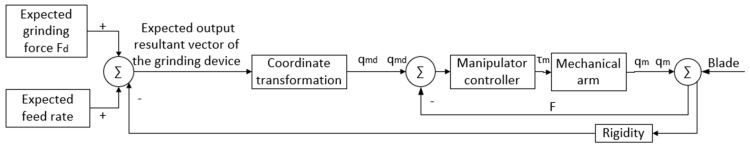
Control strategy of grinding robot applied in the grinding stage.

In the second stage of the grinding operation, due to the presence of different normal vectors at each grinding point on the outer surface of the wind turbine blade, each part of the grinding robot that can be manipulated needs to be coordinated and controlled to enable pose adjustment. Based on the Peterson equation, the empirical formula of the material removal rate during the grinding process [28,29] is

(1) $$r=C_{A}K_{A}k_{t}\frac{V_{b}}{V_{w}l_{w}}F_{A}$$

where *r* is the instantaneous removal rate of material, $C_{A}$ is a constant determined via experimentation, $K_{A}$ is the resistance coefficient, $k_{t}$ is the durability coefficient of the grinding tool, $V_{b}$ is the cutting speed, $V_{w}$ is the feed rate, $l_{w}$ is the width of the grinding area and $F_{A}$ is the grinding force. The factors that most determine the impact of the grinding robot on the blade are the grinding force and the feed rate, so these two factors should be controlled in all following steps. The control strategy applied is shown in Figure 5, and this strategy enables real-time surface tracking and the constant control of the grinding force.

**Figure 6:**
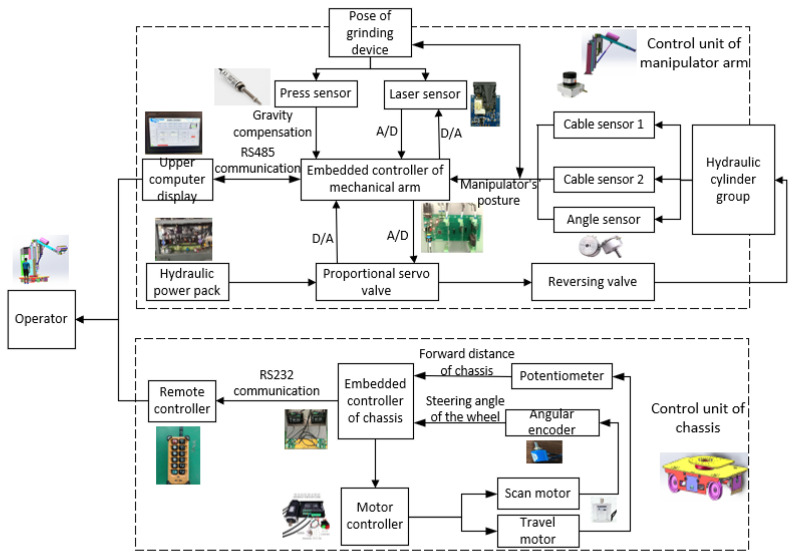
Scheme of control unit.

In summary, the control scheme applied to the grinding robot, in which the moving chassis, the manipulator and the front-end grinding device are hierarchically controlled, is shown in Figure 6. The commands controlling the chassis are sent via a remote controller, and each wheel group is controlled separately. Data on the distance and angle of movement are collected by the encoder and angle sensors and are sent back to the host computer. Information on the positioning of the manipulator and the grinding device is collected by multiple sensors and transmitted to the host computer. The two laser sensors installed on the upper and lower edges of the grinding device ensure that the tangent of the grinding roller and blade is perpendicular to the normal vector of the grinding point of the blade, and the displacement sensor included in the pose adjustment system described above controls the pressure of the grinding device on the blade. Cable sensor 1 on the lifting part of the manipulator, the angle sensor incorporated in the angle adjustment mechanism, and cable sensor 2 on the telescopic part jointly control the grinding force and speed of the robot.

## 3. Control Strategy of Grinding Robot

In the above analysis, the grinding robot is divided into two parts: the manipulator and the moving chassis, the latter of which can be controlled by the human operator. Therefore, control of the chassis is not addressed in detail in the subsequent discussion, wherein we consider the real-time tracking of the blade’s surface and the maintenance of consistent grinding via the control of the manipulator.

### 3.1. Surface Tracking Control Strategy

#### 3.1.1. Surface Tracking of Blade Surface

**Figure 7:**
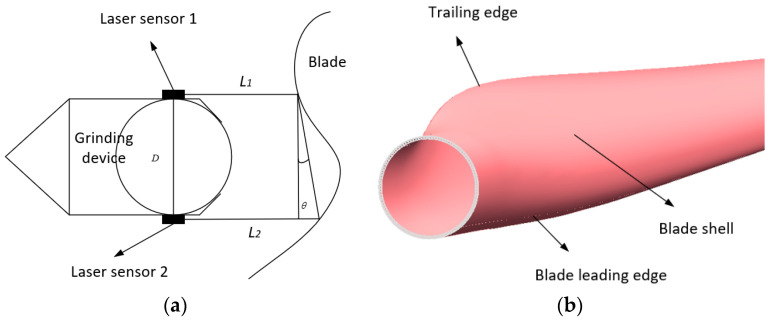
Surface tracking diagram. (**a**) Surface tracking method; (**b**) Different grinding areas of blade.

Due to the changing curvature and irregular shape of wind turbine blades, it is very important for a grinding robot to track its position on a blade’s surface during grinding, and so it is necessary to establish a reasonable control strategy [30]. Two PANFEE L2S laser ranging sensors are included above and below the front-end polishing device to track the blade surface, as shown in Figure 7a.

Here, the parameters $L_{1}$ and $L_{2}$ represent the distance between the grinding device and the surface of the wind turbine blade as measured by the laser sensors above and below the grinding device, respectively; *D* represents the distance between the two laser sensors, and *θ* represents the angle of offset between the attitude of the grinding device and the deviation of the blade, the calculation formula of which is

(2) $$\;\theta =\;\arctan\left(\frac{L_{2}-L_{1}}{D}\right)$$

After calculating θ, the pitch angle of the grinding device can be adjusted using fuzzy PID control until θ reaches zero and the parameters $L_{1}$ and $L_{2}$ are equal, which ensures that the grinding device is parallel to the tangent line of the blade’s surface at the point of grinding, and this process is carried out in real time during grinding.

In addition, when the two relevant degrees of freedom—of the three degrees of freedom of lifting, stretching and angle adjustment in the manipulator—are determined, the end of the grinding roller can be moved along any trajectory. Therefore, in order to optimize the control algorithm, the position surface tracking of the robot is planned according to the three limit conditions in the grinding process, as shown in Figure 7b.

Firstly, when the grinding area is the trailing edge of the blade, the grinding surface is almost an inclined plane and the curvature change is comparatively small. Therefore, combining the two degrees of freedom of lifting and stretching, the robot can realize the grinding work at the trailing edge of the blade.

Secondly, when the grinding area is the middle shell, the curvature of the blade begins to change significantly, and the effect of the telescopic manipulator is small, so the degrees of freedom of lifting and angle adjustment are used at this time.

Finally, when the grinding area is the leading edge of the blade, the surface shape is convex, and it is also the bottom end of a single grinding process. At this time, the lifting degree of freedom no longer applies; in consequence, combining the two degrees of freedom of stretching and angle adjustment, the grinding action can satisfy the surface of the leading edge of the blade.

In this way, the tracking of blade surface in a single grinding process can be realized.

#### 3.1.2. Adjustment of the Robot’s Position

**Figure 8:**
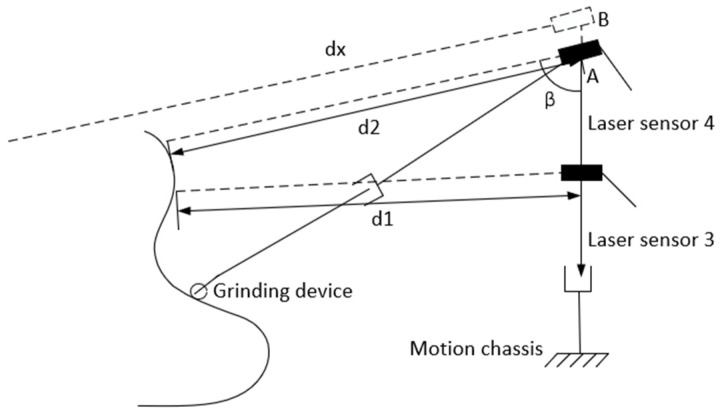
Detection of the blade’s highest edge.

To ensure the safe operation of the grinding robot and improve the overall performance, the grinding robot must keep a safe vertical distance from the blade (set at about 1.5~2.5 m in this paper), which is measured using laser sensor 3, shown in Figure 8. In order to prevent the lift height of the robot’s grinding device from exceeding its stroke distance, the top edge of the blade should be monitored to prevent the grinding device from sticking and causing failure. In this paper, two laser sensors are included at different heights on the lifting manipulator. Laser sensor 3 is set horizontally to detect the horizontal distance between the grinding robot and the wind turbine blade. Laser sensor 4 is attached at the top of the grinding robot to detect the distance between the robot and the highest point of the blade.

Here, $d_{1}$ is the vertical distance between the grinding robot and the real-time position of the blade, β is the angle of laser sensor 4, $d_{2}$ is the distance measured by laser sensor 2, and $d_{x}$ is the distance as roughly calculated from angle β and $d_{1}$, the relevant formula for which is

(3) $$\;d_{x}=\frac{d_{1}}{\sin\beta }$$

$d_{x}$ as calculated by Equation (3) is compared with the distance $d_{2}$ measured by laser sensor 4. If $d_{2}$ is much larger than $d_{x}$, the grinding robot has reached the highest edge of the wind turbine blade; when $d_{2}$ gives the distance from laser sensor 2 to the ground, then the lift manipulator is commanded to stop moving and rise no further.

**Figure 9:**
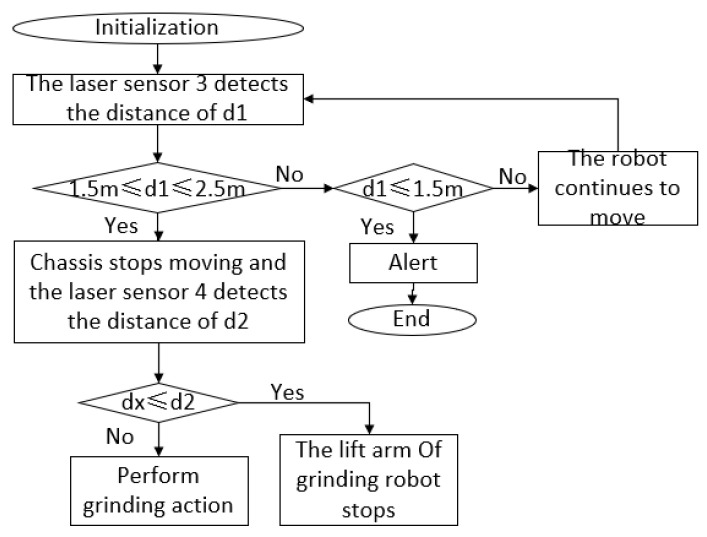
Flowchart showing the safe position control of the grinding robot.

Figure 9 shows the control process of ensuring the grinding robot’s safe positioning. Firstly, it is determined whether the vertical distance *d*_1_ between the robot and the blade, as measured by laser sensor 3, is within the safe distance of 1.5~2.5 m. If it is within this range, the chassis stops moving. Then the distance $d_{2}$ is compared with $d_{x}$, and if $d_{2}\gg d_{x}$, then the lift manipulator stops rising; otherwise, it commences the grinding action. If $d_{1}\leq 1.5\;m$, the robot is alerted and will stop moving forward. If $d_{1}\geq 2.5\;m$, the robot will continue to move closer to the wind turbine blade until it reaches a safe distance.

### 3.2. Constant Force Grinding Control Strategy

**Figure 10:**
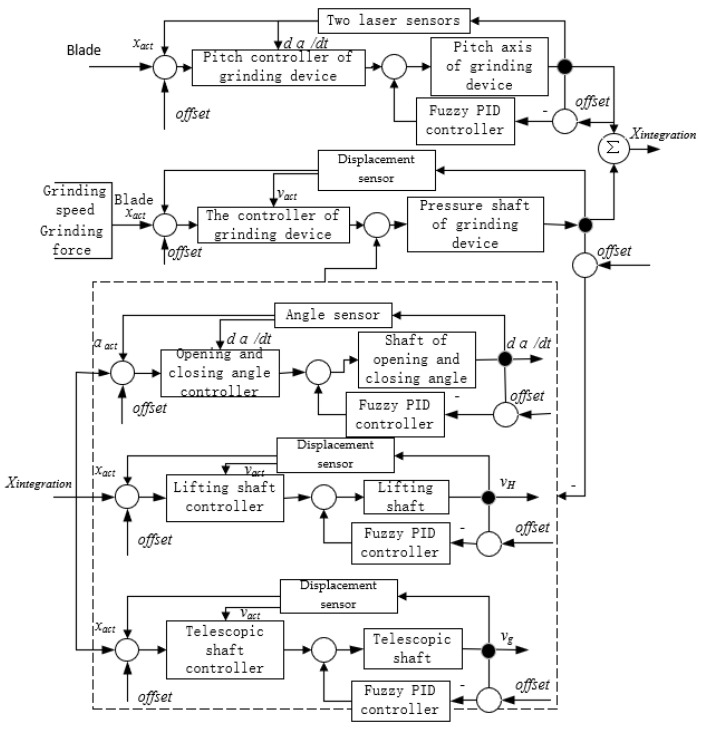
Five-axis synchronous control strategy diagram.

According to the above analysis, the strategy used for controlling the grinding force and feed speed of the device is the key to realizing the constant control and compliance of the force of grinding. There are five degrees of freedom between the manipulator and the grinding device of the robot. The synchronous control strategy is illustrated in Figure 10, the ideal output of which is the hydraulic cylinder that controls the driving manipulator and the servo motor that drives the grinding device. In order to improve the response speed of the hydraulic system, fuzzy PID is used to control each hydraulic cylinder in the manipulator.

**Figure 11:**
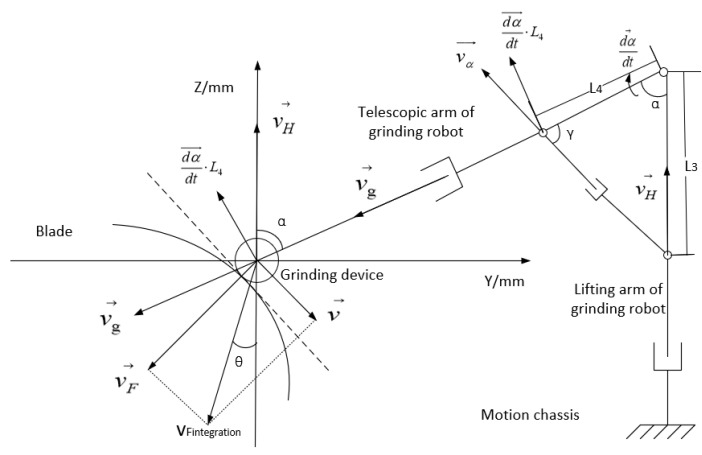
Diagram of the control strategy for grinding force and speed.

In the control strategy, the input variable used to control the grinding device is its tangential angle from the surface being processed, and the grinding speed and direction of the grinding force are the final outputs. The input variables used to control the grinding force are the blade parameters and the preset grinding force, and the final output is the grinding speed and the grinding force. The control of the input variable related to the manipulator is the final output of the former two inputs; the controller outputs the running speed of each part of the manipulator in order to maintain the grinding force and grinding speed required. Using the methods described above, the relevant control strategies for adjusting the grinding speed and the direction of grinding force are adjusted. Figure 11 shows the analysis method used to control the grinding speed and the grinding force.

Here, $v_{H}$ and $v_{g}$ are the lifting speed of the lifting arm and the telescopic speed of the telescopic arm, respectively, and $v_{\alpha }$ is the speed of the hydraulic cylinder used for angle adjustment, which is not equal to the actual angular rotational velocity of the manipulator. Their corresponding relationship is

(4) $$\;\frac{d\alpha }{dt}L_{4}=v_{\alpha }\sin\gamma$$

where $\frac{d\alpha }{dt}$ is the angular velocity of the opening and closing of the mechanical arm and represents the actual speed of the grinding device; $L_{3}$ and $L_{4}$, respectively, represent the distances between the installed position of the hydraulic cylinder used for angle adjustment and the rotational center of the manipulator being adjusted; α is the angle between the lifting and telescopic manipulators; and γ is the angle between the telescopic manipulator and the hydraulic cylinder used to adjust the angle. α can be measured in real time by the angle sensor, and γ can be obtained via the following relationship:

(5) $$\gamma \;=\;\arctan\left(\frac{L_{3}\sin\alpha }{L_{4}-L_{3}\cos\alpha }\right)$$

Combining Equations (4) and (5), to meet the required opening and closing speed of the manipulator, the speed provided by the hydraulic cylinder for angle adjustment must be

(6) $$v_{\alpha }=\frac{d\alpha }{dt}\cdot \frac{L_{4}}{\sin\left[\arctan\left(\frac{L_{3}\sin\alpha }{L_{4}-L_{3}\cos\alpha }\right)\;\right]}$$

The grinding device adjusts the pressure applied on the blade by changing the displacement *l*, which is measured by the micro displacement sensor installed in the pose adjustment device. The resultant vector of the differentiated feed speed $v_{F}$ and the stretching speed of the grinding device must both be adjusted in real time to ensure the correct grinding force, as shown by the following equation:

(7) $$\vec{v_{F}{}_{integration}}=\vec{v_{F}}+\vec{v}=\frac{\vec{dl}}{dt}+\vec{v}$$

The mathematical relationship between the coordinate systems of the grinding robot and those of each joint is essentially nonlinear, and can be expressed by the following nonlinear vector value function:

(8) x(t)=f[q(t)]

So, the operating speed ultimately required is given by

(9) $$\dot{x}={\left[\dot{x_{1}},\dot{x_{2}},\cdots \dot{x_{m}}\right]}^{T}=v_{integration}$$

To provide the speed ultimately required, the speed required from each joint is

(10) $$\dot{q}={\left[{\dot{q}}_{1},{\dot{q}}_{2},\cdots {\dot{q}}_{n}\right]}^{T}$$

The relationship between the required speed of the end and the speed of each link can be obtained by applying a kinematic calculation to the robot, which is shown in the following expression:

(11) $$\dot{x}=J\dot{q}$$

where J is the Jacobi matrix of the robot, the specific value of which can be derived from the kinematics of the grinding robot, as shown in the following expression:

(12) JT(q)=[JT1(q) JT2(q) JT3(q) JT4(q) JT5(q)]

The elements in the formula are

(13) JT1(q)=[0(d2−d4cθ3)(cθ5sθ3−cθ3sθ5)+(d4sθ3+a2)(sθ3sθ5−cθ3cθ5)d4sθ3+a2cθ5sθ3−cθ3sθ5−sθ3sθ5−cθ3cθ50]JT2(q)=[00−1000]JT3(q)=[0−d4sθ50sθ5cθ50]JT4(q)=[001000]JT5(q)=[000001]

The above-designed control strategy ensures that the correct speed is provided by each connecting rod at any given time in order to meet the grinding requirements, as

(14) $$q\left(t\right)=J^{-1}\left(q\right)\dot{q}\left(t\right)$$

In order to resolve the problem whereby the inverse Jacobian matrix of the grinding robot does not exist because of the redundancy of the mechanical arm, the following expression can be used [31]:

(15) $$C=\frac{1}{2}{\dot{q}}^{T}A\dot{q}+\lambda^{T}\left[\dot{x}-J\left(q\right)\dot{q}\right]$$

where C is an m × m symmetric positive definite matrix, and λ is the cost judgement. By minimizing C, the speed of each link and λ, we can obtain the following expression:

(16) $$\dot{q}\left(t\right)=A^{-1}J^{T}\left(q\right)\lambda$$

(17) $$\dot{q}\left(t\right)=J\left(q\right)\dot{q}\left(t\right)$$

By combining the above two expressions, λ can be derived as

(18) $$\lambda ={\left[J\left(q\right)A^{-1}J^{T}\left(q\right)\right]}^{-1}\dot{x}\left(t\right)$$

By combining Equations (16) and (18), the desired speed x(t) is given for the end of the grinding device, and the speed of each joint is obtained according to Equation (18):

(19) $$\dot{q}\left(t\right)=A^{-1}J^{T}\left(q\right){\left[J\left(q\right)A^{-1}J^{T}\left(q\right)\right]}^{-1}\dot{x}\left(t\right)$$

where *A* is an identity matrix. Equation (19) can be simplified to Equation (14), which relates the grinding blade’s position and time. The position of the end of the grinding device, which is related to the sample taken at any given time, is a fixed value, and the sampling period is 200 ms.

**Figure 12:**
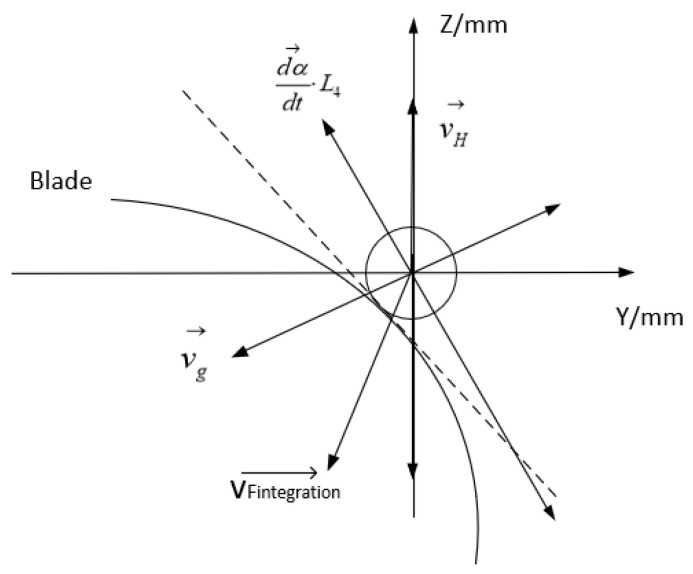
Instantaneous velocity relationship of contact force control strategy.

Due to the redundancy of the grinding robot, there are multiple solutions to determining the expected speed of the robot end at any time. In order to ensure optimal control of the robot, the change in speed of the connecting rod must be as small as possible in any sampling period. Using the sensor and a calculation of the mathematical relationship, the angle between the desired direction of travel at the end and the direction of each connecting rod at any given time can be obtained, as shown in Figure 12.

Give its three-part structure, the robot can be moving in two directions at any given time. For example, the lifting system can be raising or lowering at any time. In order to ensure its compliance to control, the angle between its direction of motion and the desired direction of the end must be as small as possible [32]. The speeds required of each connecting rod are calculated by Equation (19). On this basis, the required driving force, or torque, which is also the force provided by the hydraulic cylinder at any time, can be obtained by the following expression:

(20) $$f_{4}=D_{44}{\ddot{q}}_{4}\left(t\right)+D_{45}{\ddot{q}}_{5}\left(t\right)+D_{433}{\dot{q}}_{3}\left(t\right){\dot{q}}_{4}\left(t\right)$$

where $D_{ijk}\left(q\right)$ is the inertial matrix of the grinding robot.

### 3.3. Control Strategy of Mechanical Arm Power System

**Figure 13:**
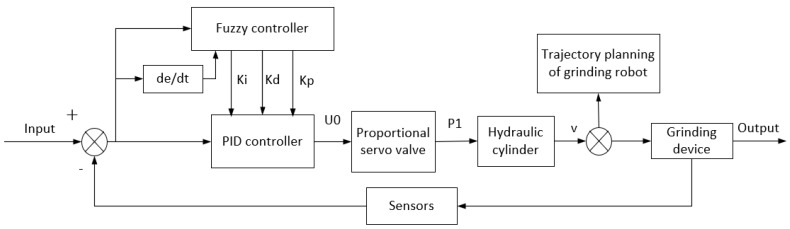
Fuzzy PID control principle.

In maintaining constant control over the force of the grinding robot, the position of the manipulator will change at any given time, so the PID parameters of the hydraulic control system need to be dynamically adjusted in real time. Based on the necessity of real-time surface tracking of the turbine blade and the application of constant force during grinding, the fuzzy PID control principle used to control the hydraulic system is shown in Figure 13.

Through the analysis of the grinding control system, it is finally determined that the inputs of the fuzzy PID control are the deviation and deviation increment of the combined vector of the expected grinding feed speed and the expected grinding force and the combined vector of the two vectors at any time, which is used as the input to control the adjustment of the fuzzy parameters *K_p_*, *K_i_* and *K_d_* in real time.

#### 3.3.1. Fuzzy Input Value

**Figure 14:**
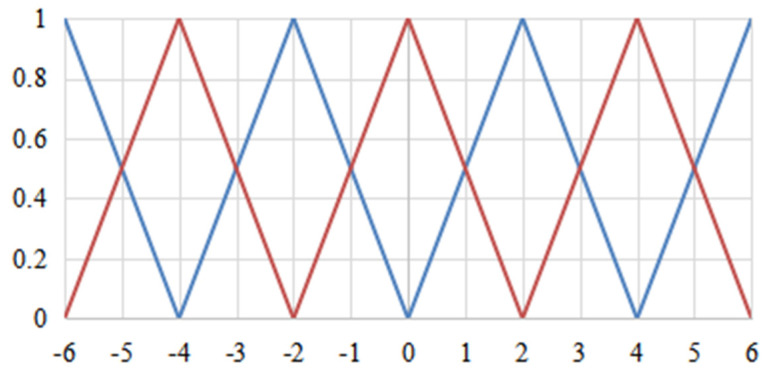
Membership function of fuzzy controller.

In order to achieve fuzzification, it is necessary to fuzzify the calculated values. In this design, the fuzzy subset is {NB, NM, NS, ZO, PS, PM, PB}, the domain is {−6, −5, −4, −3, −2, −1, 0, 1, 2, 3, 4, 5, 6}. There is an interval for the measured force and velocity signals, which is made as same as the PID adjustment time. Deviation and deviation change rate are shown as:

(21) $$f\left(e\right)=\frac{6\cdot e}{V_{max}-V_{min}}$$

(22) $$f\left(e\right)=\frac{6\cdot ec}{2\left(V_{max}-V_{min}\right)}$$

where *e* is the deviation and *ec* is the deviation increment. The quantization of input error and error change rate can be obtained by using the above equation. The functional relationship of the linear membership function is shown in Figure 14.

#### 3.3.2. Establish Fuzzy Rule Table

After modeling the input, *K_p_*, *K_i_* and *K_d_* in the PID control are adjusted. The specific fuzzy rules are shown in Table 1. According to the fuzzy rule table and the deviation and its change rate calculated by the value collected by the sensor and the expected value, the fuzzy subsets corresponding to ∆*K_p_*, ∆*K_i_* and ∆*K_d_* are obtained.

#### 3.3.3. Demoulding Processing

Since *K_p_*, *K_i_* and *K_d_* are adjusted in the actual fuzzy control, it can be quantified by using the center of gravity calculation according to the triangular membership function, as shown in Equation (23).

(23) $$v_{0}=\frac{\sum_{i=0}^{n}M_{i}F_{i}}{\sum_{i=0}^{n}M_{i}}$$

where *M* is membership and *F* is fuzzy quantization value. The sum of membership is 1, so the denominator can be omitted, so that the calculation of each object is actually a matrix operation:

(24) $$K=\left[M_{e1}\;M_{e2}\right]\left[\begin{array}{c} F_{a}\;F_{b}\\ F_{e}\;F_{d} \end{array}\right]{\left[M_{ec1}\;M_{ec2}\right]}^{T}$$

Coefficients can also be introduced to enlarge and reduce the variation of *K_p_*, *K_i_* and *K_d_* after the increment is obtained.

**Figure 15:**
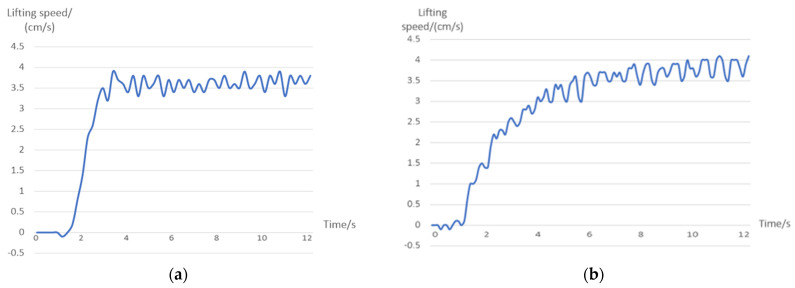
The performance indices. (**a**) with fuzzy PID control and (**b**) without fuzzy PID control.

(25) K(n)=K(n−1)+ΔK·λ

where λ is set to 1 in this control strategy and ΔK is the calculated value. The hydraulic cylinder driving the lifting manipulator is taken as an example for this experimental analysis. The performance indices of the hydraulic cylinder with fuzzy PID control and without fuzzy PID control are compared, as shown in Figure 15, and their specific indices are shown in Table 2.

It can be seen that, when using fuzzy PID control, the hydraulic cylinder shows a small degree of overshoot and fast response, and the errors arising from the designed static control strategy can be greatly reduced.

### 3.4. Analysis of Gravity Compensation

**Figure 16:**
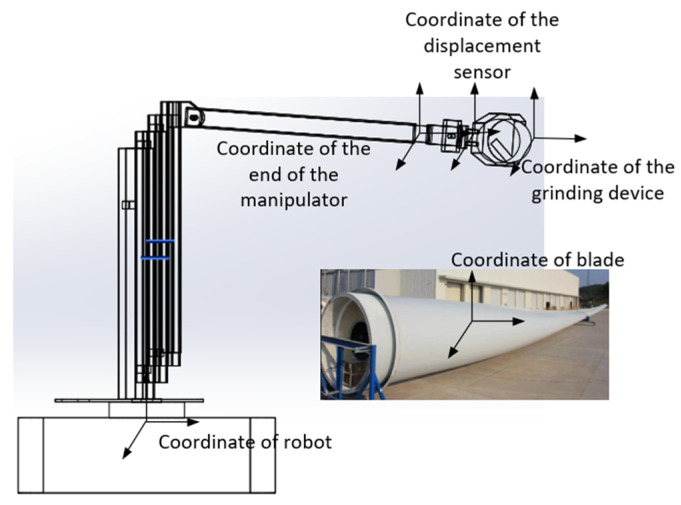
Diagram of relative positional coordinates.

The grinding device of the robot described in this paper is attached to the end of the manipulator by a fixed connection, and a schematic diagram of the robot’s coordinates of movement is shown in Figure 16. In addition to the parameters relevant to the control strategy, the factors influencing the stability of the control strategy also include the contribution of the grinding device’s own gravity to the force applied.

**Figure 17:**
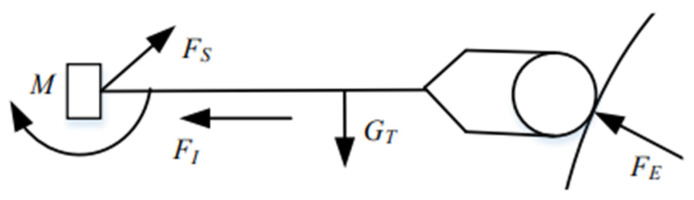
Analysis diagram of force measured by sensor.

The overall force includes the contact force $F_{E}$, the gravity of the grinding device $G_{T}$ and the inertial force of acceleration during the movement of the manipulator $F_{I}$, so the force measured by the sensor cannot be directly used in the algorithm. The influence of inertial force can be ignored because the acceleration of the manipulator is negligible, and gravity compensation can be incorporated to eliminate the influence of the grinding device’s gravity. Figure 17 shows the analysis of the force measured by the sensor in the grinding device, and the relation is shown in Equation (26).

(26) $$\vec{F_{S}}=\vec{F_{E}}+\vec{G_{T}}+\vec{F_{I}}$$

This can be simplified as

(27) $$\vec{F_{S}}=\vec{F_{E}}+\vec{G_{T}}$$

Using the rotation transformation matrix of the robot, the force of the gravity of the grinding device as measured by the sensor can be expressed as

**Figure 18:**
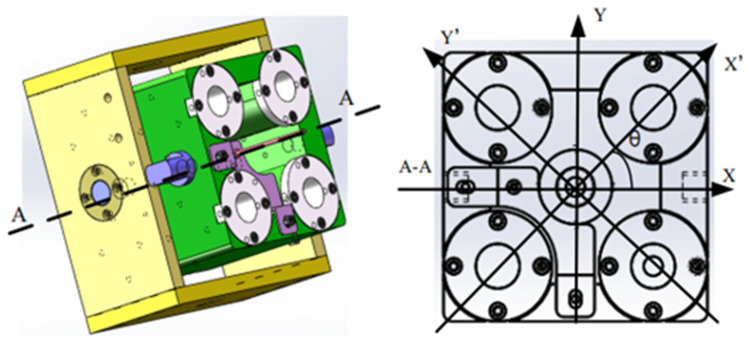
The grinding device and the sensor’s coordinate systems.

(28) GTS=RESROEGTO

where GTS is the gravitational force vector of the front-end grinding device in the coordinate system given by the displacement sensor, RES is the rotation matrix derived from the system of axes of the terminal part of the robotic arm in relation to the sensor’s coordinate system and ROE is the rotation matrix of the frame of the axes of the grinding device in relation to the basal coordinate system, the relative expression of which is

(29) $${}_{O}^{E}R=\left[\begin{array}{ccc} c\theta_{1}\left(c\theta_{3}c\theta_{5}+s\theta_{3}s\theta_{5}\right) & c\theta_{1}\left(s\theta_{3}s\theta_{5}-c\theta_{3}s\theta_{5}\right) & -s\theta_{1}\\ s\theta_{1}\left(s\theta_{3}c\theta_{5}+s\theta_{3}s\theta_{5}\right) & s\theta_{1}\left(s\theta_{3}c\theta_{5}-c\theta_{3}s\theta_{5}\right) & c\theta_{1}\\ s\theta_{3}c\theta_{5}-c\theta_{3}s\theta_{5} & -s\theta_{3}s\theta_{5}-c\theta_{3}s\theta_{5} & 0 \end{array}\right]$$

where $\theta_{i}$ (i = 1~5) is the angle of the joints between mechanical arms I to V. There is a fixed deflection θ between the fixed system of axes of the grinding device and the system of axes of the sensor used to measure the pressure, as shown in Figure 18.

The rotational transformation of the system of axes of the sensor relative to the flange’s coordinate system is

(30) RES=[cθ−sθ0sθcθ0001]

Combining the above four formulas, the following expression can be obtained:

(31) $$[\begin{array}{c} F_{x}\\ F_{y}\\ F_{z} \end{array}]=\left[\begin{array}{ccc} c\theta & -s\theta & 0\\ s\theta & c\theta & 0\\ 0 & 0 & 1 \end{array}\right]\cdot \left[\begin{array}{ccc} c\theta_{1}\left(c\theta_{3}c\theta_{5}+s\theta_{3}s\theta_{5}\right) & c\theta_{1}\left(s\theta_{3}s\theta_{5}-c\theta_{3}s\theta_{5}\right) & -s\theta_{1}\\ s\theta_{1}\left(s\theta_{3}c\theta_{5}+s\theta_{3}s\theta_{5}\right) & s\theta_{1}\left(s\theta_{3}c\theta_{5}-c\theta_{3}s\theta_{5}\right) & c\theta_{1}\\ s\theta_{3}c\theta_{5}-c\theta_{3}s\theta_{5} & -s\theta_{3}s\theta_{5}-c\theta_{3}s\theta_{5} & 0 \end{array}\right]\cdot \left[\begin{array}{c} 0\\ 0\\ -G_{T} \end{array}\right]$$

Simplifying Equation (31), the following expression can be given:

(32) $$cos\theta =\sqrt{\frac{F_{x}^{2}+{cos}^{2}(\theta_{3}+\theta_{5})}{{cos}^{2}(\theta_{3}+\theta_{5})+{sin}^{2}(\theta_{3}-\theta_{5})}}$$

Multiple sets of data pertaining to the installation deflection angle *θ* can be derived from information on the angles of the robot’s different poses, and an average value can be obtained. Because the micro displacement sensor is used to collect force information, the force that is measured is only unidirectional, and its calculation formula is

(33) FsE=F−GTcos(θ3−θ5)cos(θ+θ1)/cos2θ5tan2θ5+(cos2θ3+sin θ3sin θ1)+cos θ3tan θ5(sin θ3−sin θ1)

where FsE is the grinding force as a component of the coordinate system measured by the pressure sensor, and F is the grinding force calculated by the displacement sensor. In the process of grinding a given area, the rotational angle of the grinding robot is always ±90°, so Equation (34) can be simplified as

(34) FsE=F−GTcos(θ3−θ5)sinθ/cos2θ5tan2θ5+cos2θ3+cos θ3tan θ5sin θ3

The operational flow of gravity compensation in the grinding robot is as follows:

- The grinding device performs a profiling movement on the blade’s surface when it is not in contact with the blade and the grinding force is zero, after which time the measurements of the pressure sensor and the manipulator in each attitude are collected;
- The force and attitude information collected are substituted into Equation (33) to obtain the installation deflection angle;
- The above two steps are repeated to collect multiple different sets of information and thus obtain multiple installation deflection angles, which can be used to calculate the average installation deflection angle θ;
- The deflection angle obtained in the previous step is substituted into Equation (34) to determine whether the result is 0; if it is not 0, the above steps are repeated until the contact force obtained is 0.

In this way, gravity compensation can be realized; the influence of the gravitational force of the grinding device can be eliminated using the above formula, and the real contact force can thus be obtained, which can then be applied in the constant force control algorithm.

## 4. Experiments

To verify the feasibility of our control strategy, the surface tracking and constant force control of the grinding robot have been experimentally verified in a grinding operation. The experimental results of robotic grinding are here compared with the artificial results, and this verifies the feasibility of the designed control strategy, according to which the grinding robot can meet the requirements of a given processing site.

### 4.1. Control Algorithm Comparison Experiment

**Figure 19:**
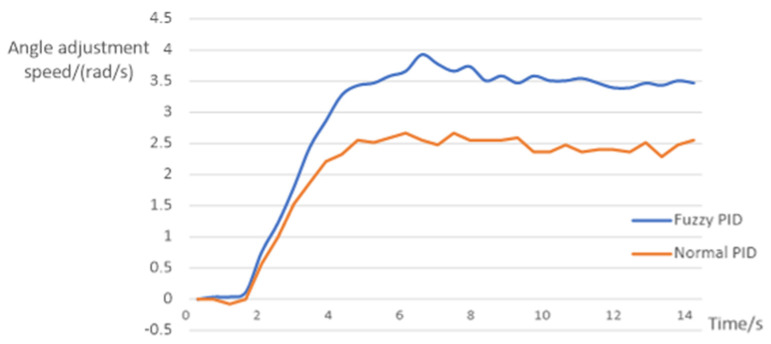
Curve of velocity response.

Taking the driving hydraulic cylinder at the opening and closing manipulator as an example, the experimental analysis is carried out. The performance indices of hydraulic cylinders using fuzzy PID control and normal PID control when the length of the telescopic arm is 218.5 mm are compared as shown in Figure 19. The maximum overshoot is 0.27 rad/s.

The reason for the large difference between the two response curves is that the opening and closing angle corresponds to the expansion length of the hydraulic cylinder. Normal PID has different control effects when the hydraulic cylinder telescopic length is different. However, the three parameters *K_p_*, *K_i_* and *K_d_* of fuzzy PID are variable, which can adapt to the different lengths of hydraulic cylinder. The experiment shows that fuzzy PID control is more adaptable and superior than normal PID control.

### 4.2. Surface Tracking Experiment

This surface tracking experiment uses time intervals of 200 ms. The coordinate sizes of the tangent point of the grinding device and the blade relative to the base coordinates of the grinding robot are calculated using data given by the five laser sensors installed on the manipulator, enabling us to monitor the actual trajectory of the blade.

**Figure 20:**
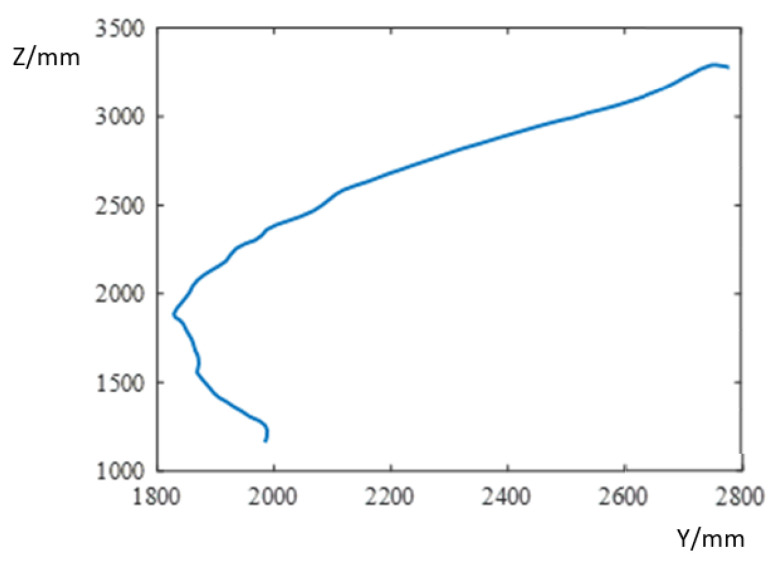
Trajectory of grinding cutting point.

In a given grinding area, the grinding robot does not change direction on the x-axis, and this value is always 0. The formulae for calculating the coordinates of the tangent point of the robot and the wind turbine blade in the y-direction and z-direction are

(35) $$y\;=d_{4}\sin\left(\frac{\pi \theta_{3}}{180^{{}^{\circ}}}\right)+600\cos\left(\pi \frac{\theta_{3}+\theta_{5}-180^{{}^{\circ}}}{180^{{}^{\circ}}}\right)$$

(36) $$z\;=d_{2}-d_{4}\cos\left(\frac{\pi \theta_{3}}{180^{{}^{\circ}}}\right)+600\sin\left(\pi \frac{\theta_{3}+\theta_{5}-180^{{}^{\circ}}}{180^{{}^{\circ}}}\right)$$

where $d_{2}$ is the position of the lifting manipulator, $d_{4}$ is the telescopic length of the telescopic manipulator, $\theta_{3}$ is the opening and closing angle of the mechanical arms, and $\theta_{5}$ is the pitch angle of the polishing device. The calculated trajectory of the cutting point coordinates in a grinding area is shown in Figure 20, and they are shown to be very close to the contours of the processing area, which verifies the utility of this control strategy for surface tracking during grinding.

### 4.3. Grinding Force Tracking Experiment

**Figure 21:**
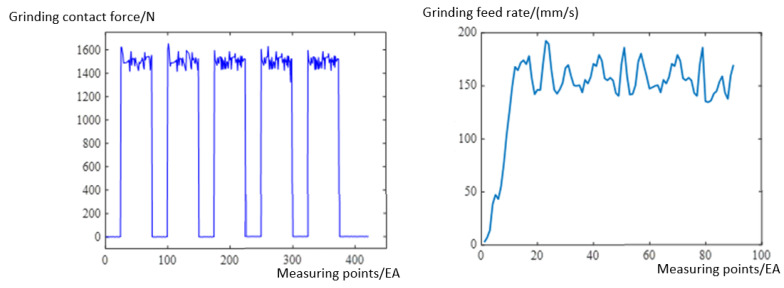
Feedback values of grinding force and grinding feed speed.

The expected values of the grinding force and feed speed are set to 1500 N and 150 mm/s, respectively, and these are compared with data collected for a grinding area at sampling periods of 200 ms. Other factors affecting the grinding are set to constant values, such as the revolution speed of the grinding device, which is 1500 r/min. Figure 21 shows the grinding contact force and feed speed, collected from top to bottom during the grinding of a processing area. The grinding contact force is maintained at about 1500 N, and the grinding feed speed remains basically stable at about 150 mm/s after contact, which verifies the utility of our strategy.

### 4.4. Field Processing Experiment

**Figure 22:**
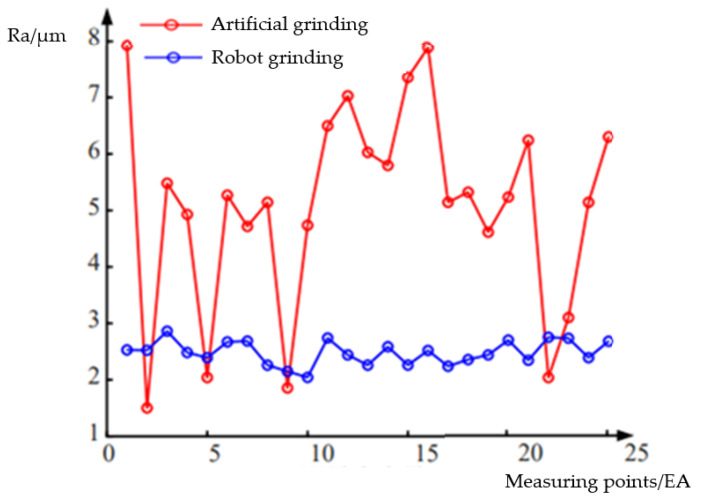
Grinding surface roughness.

Actual processing experiments of the grinding robot also needed to be carried out to verify that it could meet the needs of field processing. In the artificial grinding area and the robot grinding area, two square areas with side lengths of 500 mm were randomly selected. Within these areas, detection points were set every 100 mm along the axial direction of the grinding device and the grinding feed direction to measure the roughness of the machined surface, and the measured data are shown in Figure 22. The average roughness yielded by artificial grinding was 5.0934 μm. The average roughness yielded by robot grinding was 2.4828 μm, and all measurement points yielded data that met the surface roughness requirements for an optimal subsequent painting process.

## 5. Discussion

In this paper, we introduced a robot that can be used for grinding the surface of wind turbine blades, and we proposed related control and motion planning algorithms; we found that tracking the blade’s surface and employing the constant force control algorithm improved the grinding effect. Fuzzy PID control of the power source, focusing on the hydraulic cylinder of the manipulator, was carried out, and this greatly improved the response speed and reduced errors in the static control strategy. In order to eliminate the impact of the weight of the grinding device on the information collected regarding grinding force, a gravity compensation method for the grinding device was designed.

The experiments show that fuzzy PID has excellent adaptability and can meet the design requirements. The effect of tracking the blade’s surface during the grinding process is very impressive, enabling the average grinding force and grinding feed speed to be maintained at near the predicted values. In the actual processing experiment, the surface qualities of blades after robot grinding and artificial grinding were compared. The mean roughness of the blade after grinding by robot was Ra = 2.4828 μm, which is within the optimal range of Ra = 2.0~3.0 μm, and this makes the quality of the blade surface benefit the subsequent painting process.

Future work should study the joint motion of the chassis and the manipulator of the grinding robot. In addition to the grinding force and feed speed, other factors that influence the grinding effect, such as the grinding speed and the amount of sandpaper in the grinding device, can also be further studied.

## Figures and Tables

**Table 1:** Fuzzy control rules table.

*K_p_ K_i_ K_d_*		*ec*						
		NB	NM	NS	ZO	PS	PM	PB
*e*	NB	PB NB PS	PB NB NS	PM NM NB	PM NM NB	PS NS NB	ZO ZO NM	ZO ZO PS
	NM	PB NB PS	PB NB NS	PM NM NB	PS NS NM	PS NS NM	ZO ZO NS	NS ZO PS
	NS	PM NB ZO	PM NM NS	PM NS NM	PS NS NM	ZO ZO NS	NS PS NS	NS PS ZO
	ZO	PM NM ZO	PM NM NS	PS NS NS	ZO ZONS	NS PS NS	NM PM NS	NS PS ZO
	PS	PS NM ZO	PS NS ZO	ZO ZO ZO	NS PS ZO	NS PS ZO	NM PM ZO	NB PB ZO
	PM	PS ZO PB	ZO ZO NS	NS PS PS	NM PS PS	NM PM PS	NM PB PS	NB PB PB
	PB	ZO ZO PB	ZO ZO PM	NM PS PM	NM PM PM	NM PM PS	NB PB PS	NB PB PB

**Table 2:** Comparison of specific indicators of hydraulic cylinders with different control methods.

Control Mode	Maximum Overshoot	Rise Time	Peak Time	Steady State Error	Accommodation Time
fuzzy PID control	3.58%	700 ms	1500 ms	±0.5 cm/s	3100 ms
proportional servo valve control	5.58%	1500 ms	2700 ms	±2 cm/s	5300 ms

## Data Availability

No new data were created.

## References

[1] Dai J.C., Yang X., Wen L. (2018). Development of wind power industry in China: A comprehensive assessment. Renew. Sustain. Energy Rev..

[2] Liu Y.D., Wang J. The SWOT Analysis and Countermeasure Research on the Development of Wind Power Industry in China. Proceedings of the IOP Conference Series: Earth and Environmental Science.

[3] Chen G.W., Huang X.M., Zhang L.A., Wang J.H., Liu W.S. (2021). Study on sand grinding technology of wind turbine blade surface. Acta Energ. Sol. Sin..

[4] Yan C., Chen X.L., Li G.L., Li C.L. (2021). Design and development of an efficient and adaptive grinding head system for a new type of wind turbine blade automatic grinding robot. Compos. Sci. Eng..

[5] Xu Z.L., Lu S., Yang J., Feng Y.H., Shen C.T. (2017). A wheel-type in-pipe robot for grinding weld beads. Adv. Manuf..

[6] Ma W.C. (2019). Design and Research on Constant Force Control Device for Robot Grinding. Master’s Thesis.

[7] Xiao M. (2020). Research on Constant Force Control Methods in Robot Grinding Process. Ph.D. Thesis.

[8] Zhang T., Yu Y., Zou Y.B. (2019). An Adaptive Sliding-Mode Iterative Constant-force Control Method for Robotic Belt Grinding Based on a One-Dimensional Force Sensor. Sensors.

[9] Kuo Y.L., Huang S.Y., Lan C.C. (2019). Sensorless force control of automated grinding/deburring using an adjustable force regulation mechanism. IEEE Int. Conf. Robot. Autom..

[10] Shen Y., Lu Y., Zhuang C. (2022). A fuzzy-based impedance control for force tracking in unknown environment. J. Mech. Sci. Technol..

[11] Wahballa H., Duan J., Dai Z. (2022). Constant force tracking using online stiffness and reverse damping force of variable impedance controller for robotic polishing. Int. J. Adv. Manuf. Technol..

[12] Yao M.Y., Zheng C.W., Wang Z.H., Qian J.Z., Xi Q., Kuang S.L. Research on the method of skateboard edge grinding by combination of robot and pneumatic constant force grinding device. Proceedings of the Fourth International Conference on Mechanical, Electric and Industrial Engineering (MEIE2021).

[13] Sun M.J., Guo K., Sun J. (2021). Research on robot grinding force control method. Intell. Robot. Appl..

[14] Dai S.J., Li S.N., Ji W.B., Sun Z.L., Zhao Y.F. (2021). Force tracking control of grinding end effector based on backstepping + PID. Ind. Robot. Int. J..

[15] Xu X.H., Chen W. (2021). Hybrid active/passive force control strategy for grinding marks suppression and profile accuracy enhancement in robotic belt grinding of turbine blade. Robot. Comput. Integr. Manuf..

[16] Zhang T., Yuan C., Zou Y.B. (2022). Online Optimization Method of Controller Parameters for Robot Constant Force Grinding Based on Deep Reinforcement Learning Rainbow. J. Intell. Robot. Syst..

[17] Zhao W., Xiao J.L., Liu S.J., Dou S.X., Liu H.T. (2022). Robotic direct grinding for unknown workpiece contour based on adaptive constant force control and human–robot collaboration. Ind. Robot..

[18] Wang G., Deng Y., Zhou H., Yue X. (2023). PD-adaptive variable impedance constant force control of macro-mini robot for compliant grinding and polishing. Int. J. Adv. Manuf. Technol..

[19] Han J.L., Shan X.L., Liu H.T., Xiao J.L., Huang T. (2023). Fuzzy gain scheduling PID control of a hybrid robot based on dynamic characteristics. Mech. Mach. Theory.

[20] Li D.W., Yang J.X., Zhao H., Ding H. (2022). Contact force plan and control of robotic grinding towards ensuring contour accuracy of curved surfaces. Int. J. Mech. Sci..

[21] Jia W.D., Jiang Z.F., Dai Y. (2022). Constant Force Control Method of Grinding Device. Lect. Notes Comput. Sci..

[22] Zhao X.W., Lu H., Yu W.F., Tao B., Ding H. (2022). Vision-based Mobile Robotic Grinding for Large-scale Workpiece and Its Accuracy Analysis. IEEE/ASME Trans. Mechatron..

[23] Dai S.J., Zhang W.H., Ji W.B., Zhao Y.F., Zheng H.W., Mu J.H., Li P.W., Deng R.Q. (2022). Research on constant force grinding control of aero-engine blades based on extended state observer. Ind. Robot.

[24] Xiao M., Zhang T., Zou Y.B., Chen S.Y. (2020). Robotic constant force grinding control based on grinding model and iterative algorithm. Ind. Robot.

[25] Zhang X.X. (2022). Hybrid Coutour Force/Position Control of Industrial Robot Based on Variable-Gain Iterative Learning. Ph.D. Thesis.

[26] Li J., Guan Y., Chen H., Wang B., Zhang T. (2023). Robotic Polishing of Unknown-Model Workpieces With Constant Normal Contact Force Control. IEEE/ASME Trans. Mechatron..

[27] Zhang T., Yuan C., Zou Y.B. (2023). Research on the algorithm of constant force grinding controller based on reinforcement learning PPO. Int. J. Adv. Manuf. Technol..

[28] Peterson M.B. (1980). Design considerations for effective wear control. Wear Control Handbook.

[29] Tsai M.J., Huang J.F., Kao W.L. (2009). Robotic polishing of precision molds with uniform material removal control. Int. J. Mach. Tools Manuf..

[30] Lai H.S., Wu L., Chen X.D., Yang Z.Q. (2018). Research on the force control of the robot and surface tracking with unknown parameters. Manuf. Technol. Mach. Tool.

[31] Xiong M.Q. (2020). Research on Key Techniques of Robot Force/Position Control Grinding and Polishing. Master’s Thesis.

[32] Zhang T., Xiao M., Zou Y.B., Xiao J.D. (2019). Research on robot constant force control of surface tracking based on reinforcement learning. J. Zhejiang Univ. Eng. Sci..

